# Supplementary material for: Automatic annotation of the bHLH gene family in plants
Source: BMC Genomics. 2023 Dec 15;24:780. doi: 10.1186/s12864-023-09877-2 (PMC10722790; doi:10.1186/s12864-023-09877-2)
Supplement: Supplementary file 10 — Additional file 10: Weblogos of the 27 subfamilies identified in the bait collection [file 12864_2023_9877_MOESM10_ESM.pdf]

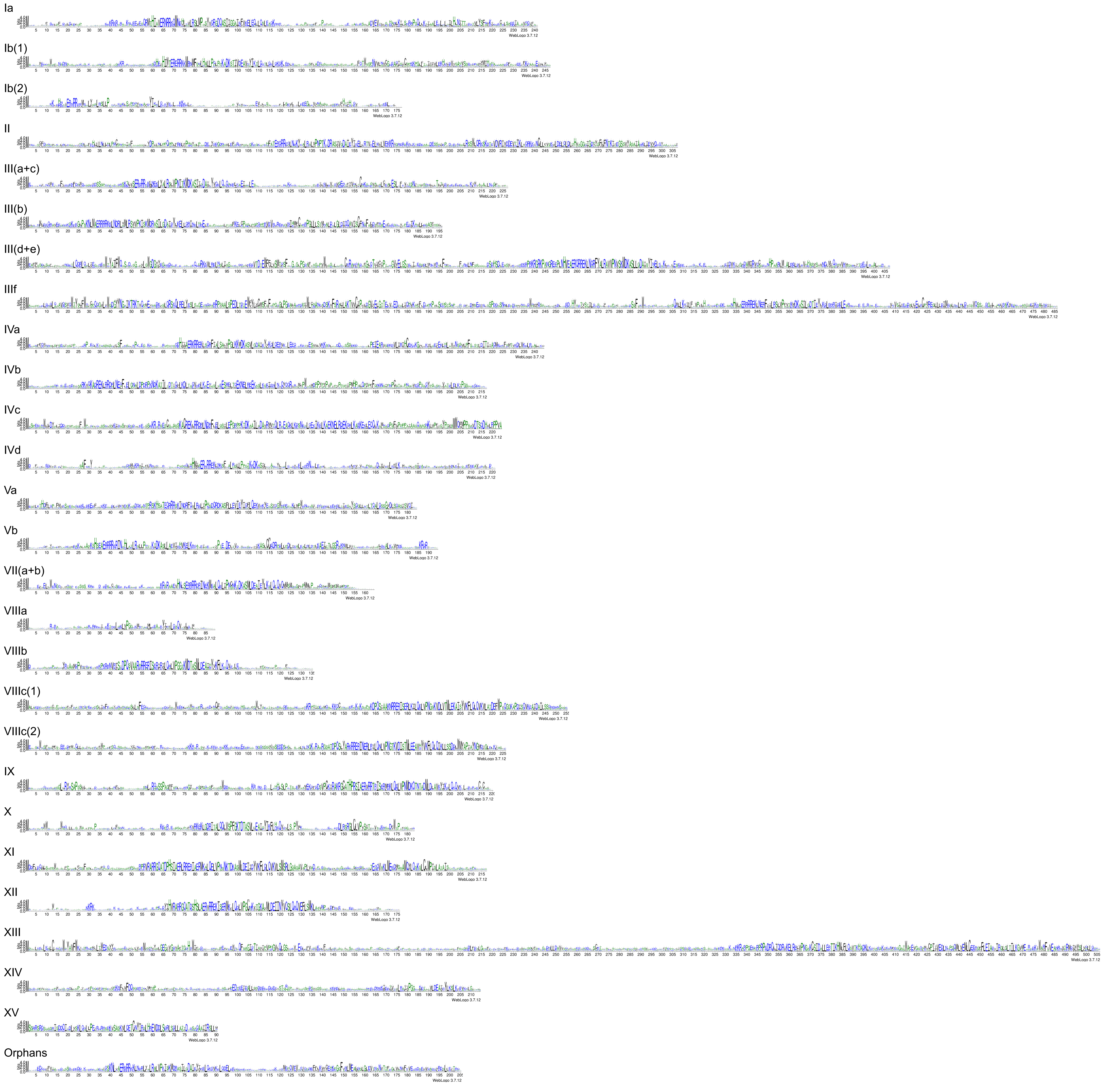

Figure S10: Weblogos of the 27 subfamilies identified in the bait collection. The subfamily member sequences were globally aligned using Muscle5 v5.1. and positions with less than 80% occupancy were removed from the alignment. Weblogos created with WebLogo 3.7.12.
