# Supplementary material for: Automatic annotation of the bHLH gene family in plants
Source: BMC Genomics. 2023 Dec 15;24:780. doi: 10.1186/s12864-023-09877-2 (PMC10722790; doi:10.1186/s12864-023-09877-2)
Supplement: Supplementary file 12 — Additional file 12: Phylogenetic tree inferred with FastTree v2.1.10 showing the phylogenetic relationship of sequences in the optimised bHLH bait collection and the optimised outgroup collection v1.1 [file 12864_2023_9877_MOESM12_ESM.pdf]

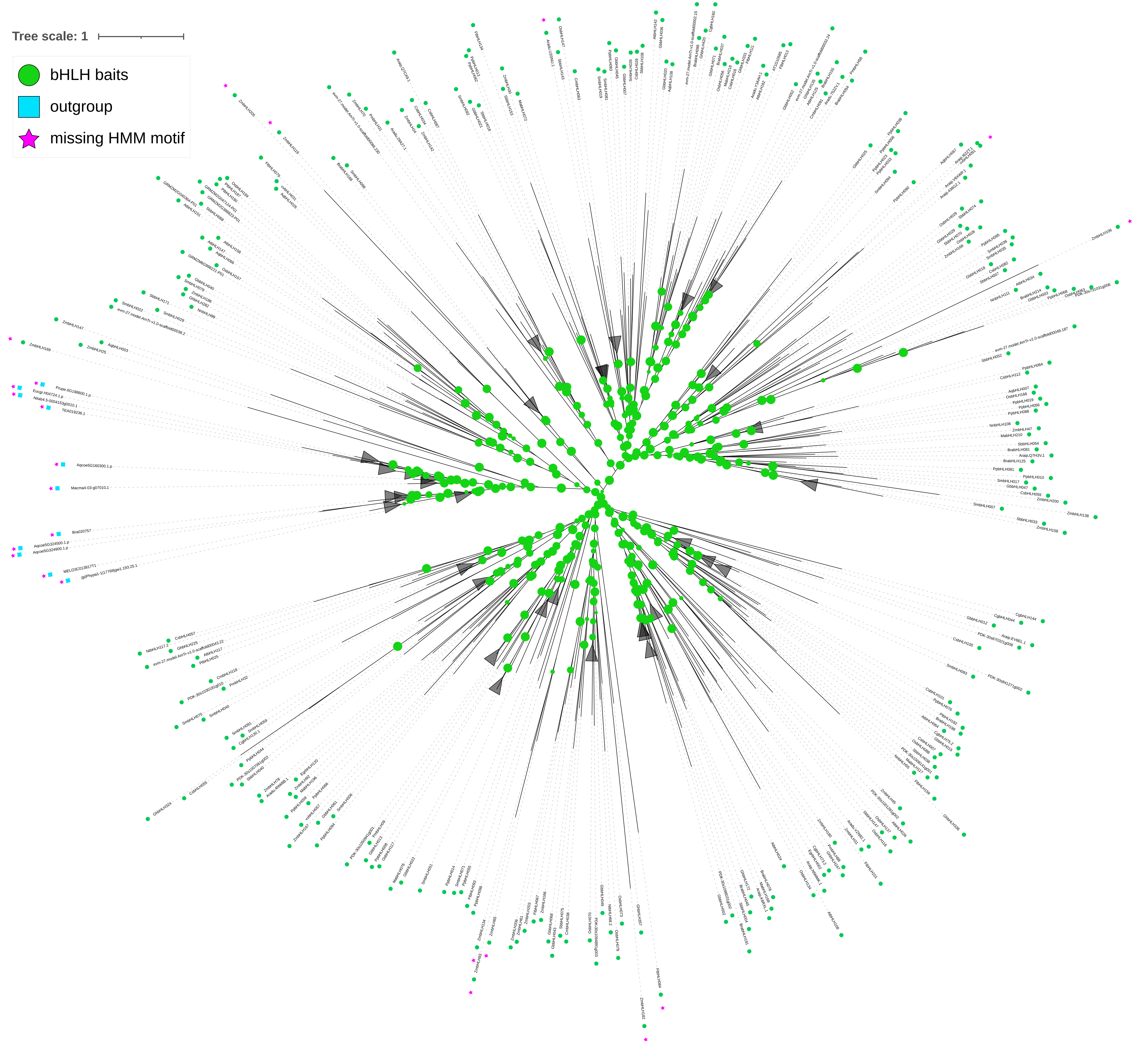

Figure S12: Maximum likelihood tree created with FastTree v2.1.10 showing the phylogenetic relationship between optimised bHLH bait collection and optimised outgroup collection v1.1. Bootstrap values are represented by the size of green circles. Clades with an average branch length distance below one are collapsed. Figure created with iTOL.
