# Supplementary material for: Automatic annotation of the bHLH gene family in plants
Source: BMC Genomics. 2023 Dec 15;24:780. doi: 10.1186/s12864-023-09877-2 (PMC10722790; doi:10.1186/s12864-023-09877-2)
Supplement: Supplementary file 13 — Additional file 13: Phylogenetic tree inferred with IQ-TREE 1.6.12 showing the phylogenetic relationship of sequences in the optimised bHLH bait collection and the optimised outgroup collection v1.1 [file 12864_2023_9877_MOESM13_ESM.pdf]

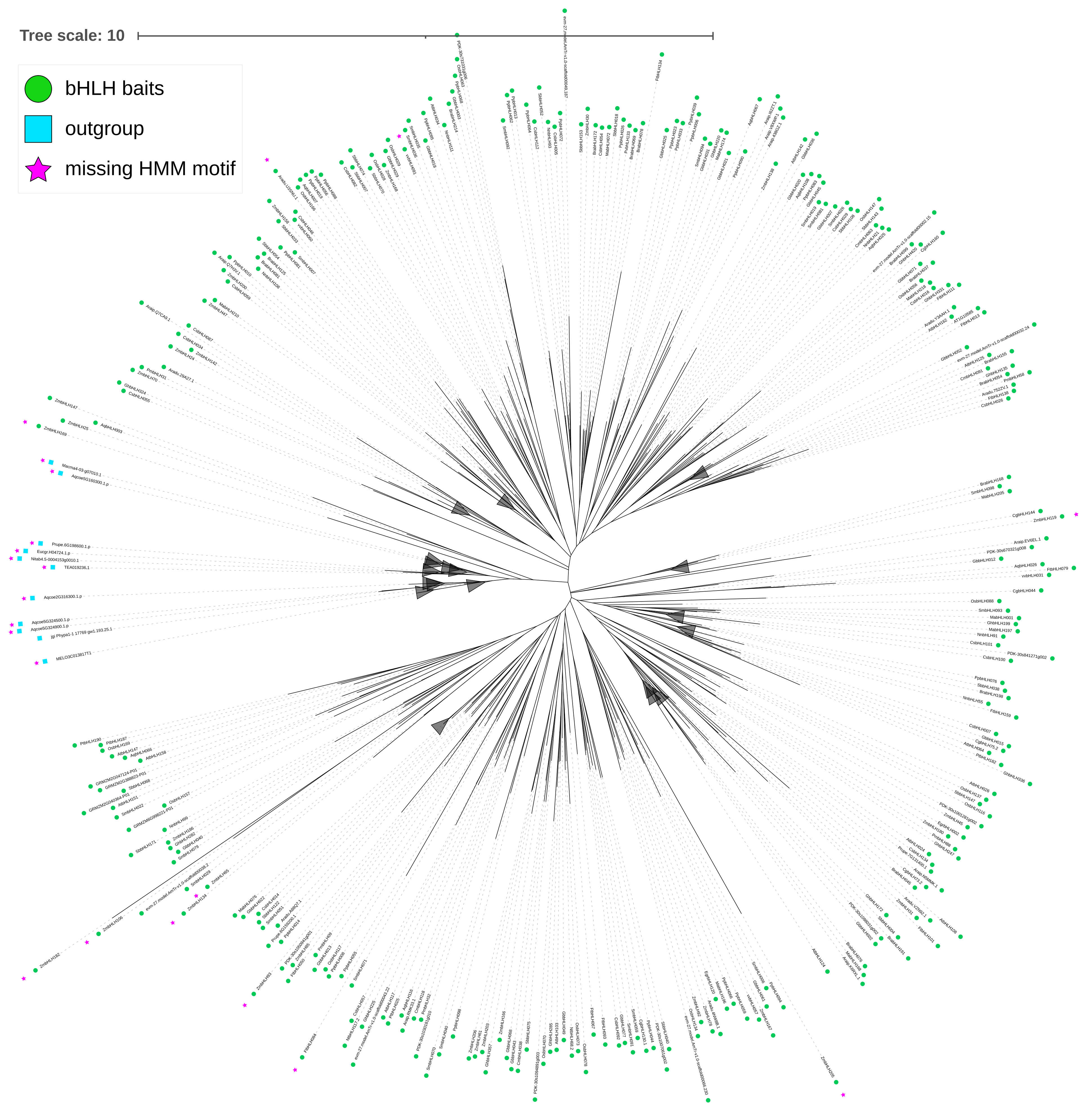

Figure S13: Maximum likelihood tree created with IQ-TREE 1.6.12 showing the phylogenetic relationship between optimised bHLH bait collection and optimised outgroup collection v1.1. Clades with an average branch length distance below one are collapsed. Figure created with iTOL.
