# Supplementary material for: Automatic annotation of the bHLH gene family in plants
Source: BMC Genomics. 2023 Dec 15;24:780. doi: 10.1186/s12864-023-09877-2 (PMC10722790; doi:10.1186/s12864-023-09877-2)
Supplement: Supplementary file 9 — Additional file 9: Phylogenetic tree displaying the twenty-seven identified subfamilies in the optimised bait collection and their representation of the land plant lineages [file 12864_2023_9877_MOESM9_ESM.pdf]

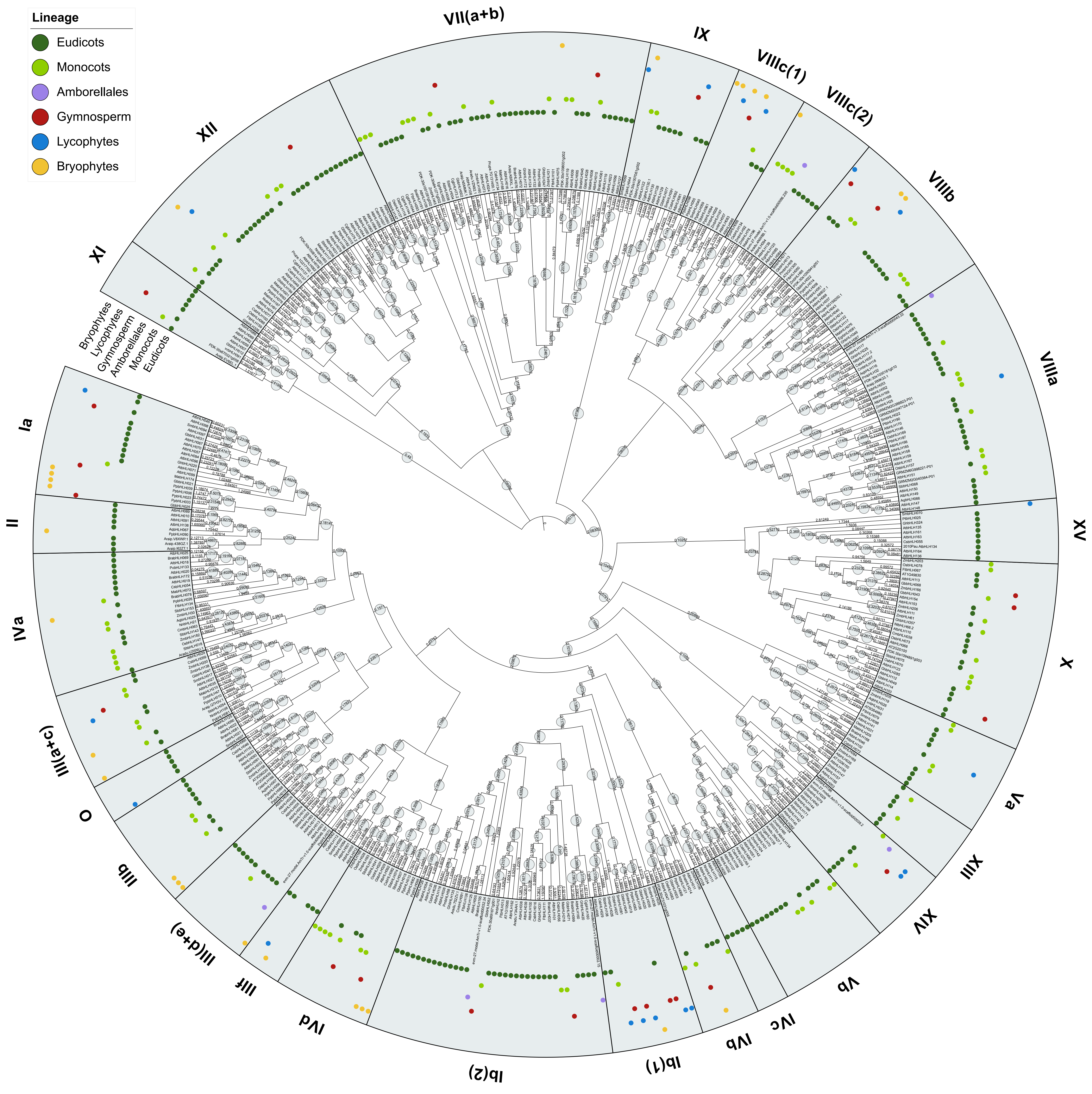

Figure S9: Maximum likelihood tree created with FastTree v2.1.10 showing the phylogenetic relationship between optimised bHLH bait collection v1.1. and *A. thaliana* bHLHs. Twenty-seven bHLH subfamilies were identified and analysed for the included land plant lineages. Branch lengths are displayed. Bootstrap values are represented by the size of the grey circles. Figure created with iTOL.
